# Case Report: Pulmonary metastases of malignant meningioma

**DOI:** 10.12688/f1000research.2-222.v2

**Published:** 2014-08-29

**Authors:** Suhail Basunaid, Frits M.E. Franssen, Ryan Accord, Myrurgia Abdul Hamid, Shekar Mahesh, Brigitta G. Baumert, Olaf E.M.G. Schijns

**Affiliations:** 1 Department of Respiratory Medicine, Maastricht University Medical Centre, Maastricht, 6202 AZ, Netherlands; 2 Department of Cardiothoracic Surgery, Maastricht University Medical Centre, Maastricht, 6202 AZ, Netherlands; 3 Department of Pathology, Maastricht University Medical Centre, Maastricht, 6202 AZ, Netherlands; 4 Department of Radiology, Maastricht University Medical Centre, Maastricht, 6202 AZ, Netherlands; 5 Department of Radiation-Oncology (MAASTRO), GROW (School of oncology), Maastricht University Medical Centre, Maastricht, 6202 AZ, Netherlands; 6 Department of Neurosurgery, Maastricht University Medical Centre, Maastricht, 6202 AZ, Netherlands

## Abstract

Meningioma accounts for approximately one-third of primary central nervous system tumors. Most meningiomas are benign, although up to one third are classified as atypical or malignant. We describe a 63-year Caucasian male presenting with pleural metastases from an intracranial meningioma. Distant metastases from meningiomas are infrequently found in clinical practice and mostly are associated with atypical or malignant meningiomas. There is no standard treatment; however surgical resection of both the primary and metastatic lesions is the safest therapy. The overall prognosis of atypical meningiomas is poor. Our patient died one week after discharge from our hospital.

## Case report

A 63-year-old Caucasian man was referred to our hospital for further analysis of slowly progressing pleural effusion with a history of cough and dyspnea. The patient had a long history of epilepsy and meningioma. He was working as head in a department of administration. He was married and had two healthy kids. As a medication he took Pantoprazol, Tegretol and Dorsolamide and had stopped smoking a long time ago.

**Figure 1.**
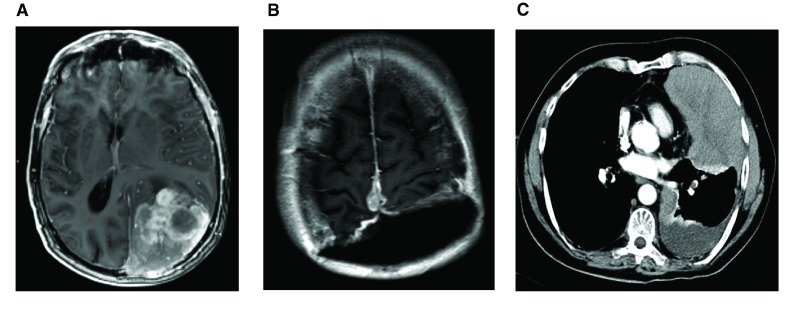
MRI and CT scanning of the original meninigioma intra-cerebral and at distance metastasis intra-pleural. **A**: T1 weighted image after administration of Gadolinium based contrast fluid shows a large extra-axial enhancing lesion in the left parieto-occipital region with local mass effect. Note that the sagittal sinus seems to be invaded. **B**: Contrast enhanced T1 weighted image after administration of Gadolinium based contrast fluid showing a large resection cavity after the second operation and recurrent disease at the most upper margin of the resection plane with enhancing areas surrounding the sagittal sinus. **C**: Midthoracal CT slice in the transverse plane. Scan performed after i.v. administration of iodine contrast. The lesion is easily distinguished at the left ventral thoracal intrapleural space, slightly enhanced suggesting solid tissue. Some pleural fluid is also present.

Our patient was diagnosed with progression of a previous operated (Simpsom ^3^ resection) and postoperatively irradiated (30 x 2 GY with a total doses of 60 Gy within the EORTC 22042 in a study context) atypical left parieto-occipital meningioma (WHO grade-II). Re-resection of the tumor (Simpsom ^4^) was performed and histopathology showed a malignant meningioma (WHO grade-III). In the follow up after re-resection there was an obvious evidence of a residual tumour at the falx cerebri. One year later an asymptomatic re-recurrence was diagnosed ( Figure 1A and B), for which conservative follow-up was performed without further surgical intervention. This was given in the form of re-irradiation with a total doses of 130 Gy (60 Gy given for the re-re-recurrent tumor at the resected area + additional 70 GY applied as an integrated boost with IMRT-technic for the residual tumor at the falx cerebri). This decision was taken due to the higher degree of aggressiveness of the malignant meningioma, as further surgical intervention would harm the patient rather than curing him.

**Figure 2.**
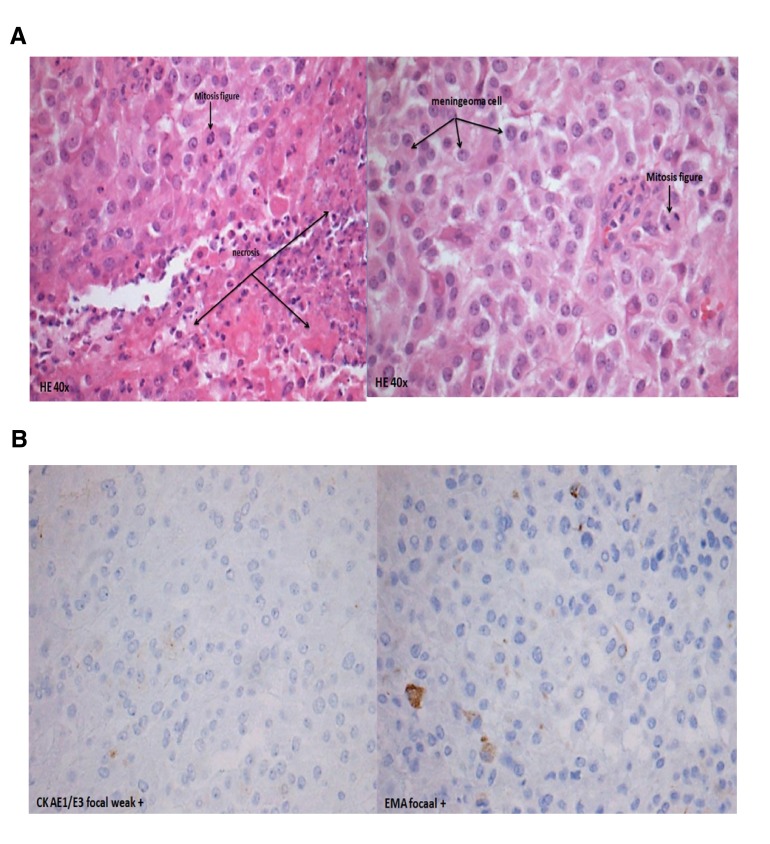
Histology staining of a biopsy of the solid intrathoracal lesion showing the same morphology as the intracranial meningioma. **A**: A specimen (HE-stained, 40x) showing histological resemblance between the intrathoracal lesion and the intracranial meningioma. **B**: Specimen (focal plus and focal weak) showed high expression in the EMA-staining and also in the AE1/AE3-staining.

A few months later the patient was hospitalized with dyspnea, fatigue, productive cough and anorexia. Multiple pleural masses were detected at a chest computer-tomography (CT) scan. Histopathology was consistent with malignant meningioma (WHO grade-III, Figure 2A), there was a high expression in the EMA staining, also in the AE1/AE3 staining ( Figure 2B). The CD 45 and CD 68 were positive and MIB-1 showed high proliferation. Palliative chemotherapy was offered but refused by the patient. The patient is died one week after discharge from the hospital as a result of voluntary euthanasia as was the will of the patient (valid written declaration).

## Discussion

Pulmonary and pleural metastases from an intracranial meningioma are very rare. Distant metastases from meningiomas are infrequently found in clinical practice and mostly associated with atypical or malignant meningiomas. Meningiomas mainly recur loco-regional or adjacent to the radiation treatment fields ^1, 2^. There are only isolated case reports regarding pulmonary metastases from meningioma. Most lung metastases were incidentally detected by chest radiography or by CT-scans, because metastatic lesions are usually asymptomatic. The presence of pulmonary metastases appears to negatively affect survival in patients with recurrent meningioma ^3, 4^.

Regarding the relationship between the intracranial location and invasion of the sagittal sinus of the tumor and the pleural metastases the route of dissemination is most probably the central venous route to heart and lungs ^5^. In previous case reports the lung was the most common extracranial metastatic site for intracranial meningioma ^6^. Our case was unusual because of the highly rate of recurrences and later the distant metastases. There is no standard treatment in the case of distant metastases.

In this case, histopathologic findings of the primary tumor revealed hypercellularity, wide necrosis, and brain invasion into the normal brain parenchyma. Pathology of the lesion from the left thoracic wall was consistent with malignant meningioma.

Other case studies described that treatment of pulmonary metastasis of malignant meningioma consisted of surgical resection for both the primary or metastatic lesions ^1, 5, 7^.

Postoperative conventional radiation therapy has been recommended for prevention of local recurrence, especially when resection is subtotal. There are insufficient data regarding radiation therapy by meningiomas with distant metastases, palliative chemotherapy is the only option in the case of distant metastases, however data regarding the efficacy of this systemic treatment are unknown.

## Informed consent

Written informed consent for publication of clinical details and clinical images was obtained from the next of kin.
