# Supplementary material for: Case Report: Pulmonary metastases of malignant meningioma
Source: F1000Res. 2014 Aug 29;2:222. Originally published 2013 Oct 21. [Version 2] doi: 10.12688/f1000research.2-222.v2 (PMC4168745; doi:10.12688/f1000research.2-222.v2)
Supplement: Peer review history

# Peer review: Referee response for version 2

**DOI:** 10.5256/f1000research.5568.r6157

**Published:** 2014-09-17

**Authors:** Christine Marosi

**Affiliations:** 1 Department of Oncology, Medical University of Vienna, Vienna, Austria

The authors added valuable information on the radiotherapy in relapse as requested, so there is one question remaining for me: was an intracranial relapse diagnosed after reirradiation? However I feel confident in approving the version 2.

I have read this submission. I believe that I have an appropriate level of expertise to confirm that it is of an acceptable scientific standard.

# Peer review: Referee response for version 2

**DOI:** 10.5256/f1000research.5568.r6093

**Published:** 2014-09-10

**Authors:** Daniel Vorobiof

**Affiliations:** 1 Department of Medical Oncology, Sandton Oncology Centre, Johannesburg, South Africa

The authors have acted on the suggestions of the reviewers and improved the description of the case. The report adds to the list of uncommon spread sites from a malignant neurological tumor.

I have read this submission. I believe that I have an appropriate level of expertise to confirm that it is of an acceptable scientific standard.

# Peer review: Referee response for version 1

**DOI:** 10.5256/f1000research.2443.r5747

**Published:** 2014-08-21

**Authors:** Antonio Santacroce

**Affiliations:** 1 Department of Neurosurgery, Heinrich Heine University, Dusseldorf, Germany

Basunaid *et al.* report on a patient harboring a recurrent meningioma who developed pleural metastases. The primary tumour is reported to be an atypical meningioma WHO Gr II, which upon recurrence shows a malignant histology WHO Gr. III.

I share the opinion of Drs Marosi and Simon about the importance of continuing reporting such clinical cases due to the rarity of such tumour entities, even though, as reported by Dr Vorobiof "It doesn't add any further information and doesn't contribute any new knowledge, neither diagnostic nor therapeutic to the current available medical literature"

There are some points which should be clarified. To quote the authors:

*"Our patient was diagnosed with progression of a previous operated and irradiated (60 GY) atypical left parieto-occipital meningioma (WHO grade-II). Re-resection of the tumor was performed and histopathology showed a malignant meningioma (WHO grade- III). After reoperation, re-irradiation (60GY + additional 70GY) was given."*

Which radiation technique was used? It is not clear whether the radiation dose applied upon recurrence was only 60Gy with the boost, and if the “additional” 70 Gy is the cumulative dosage applied including the boost, or just the boost itself.

I would also recommend verifying in the literature whether radiation therapy has been offered for histologically confirmed metastatic extracranial lesions.

I have read this submission. I believe that I have an appropriate level of expertise to confirm that it is of an acceptable scientific standard, however I have significant reservations, as outlined above.

# Peer review: Referee response for version 1

**DOI:** 10.5256/f1000research.2443.r5253

**Published:** 2014-06-27

**Authors:** Christine Marosi

**Affiliations:** 1 Department of Oncology, Medical University of Vienna, Vienna, Austria

I fully agree with the reviewer from Bonn, Matthias Simon. Meningiomas grade III are fortunately rare and they do metastasize through vascular pathways. Of course a register of such cases would be a valuable tool to get an idea of the prevalence of such events. The case reported by Basunaid is a tragic one as the patient asked for euthanasia one week after dismissal from hospital. This shows that he could not be offered any therapeutic option able to provide enough solace for staying alive.

I have read this submission. I believe that I have an appropriate level of expertise to confirm that it is of an acceptable scientific standard, however I have significant reservations, as outlined above.

# Response

**Published:** 2014-06-27

**Authors:** Suhail Basunaid

**Affiliations:** AZM/Maastricht University, Netherlands

I would like to thank Dr. Marosi for her time spent on reviewing this case report, and the valuable comments given. I would like to leave my current version as it is. I completely agree regarding the facts about the rarity of the case and the termination by the euthanasia declaration.

# Peer review: Referee response for version 1

**DOI:** 10.5256/f1000research.2443.r2861

**Published:** 2014-01-16

**Authors:** Matthias Simon

**Affiliations:** 1 Department of Neurology, University of Bonn, Bonn, Germany

Basunaid *et al.* describe a patient with a recurrent meningioma who ultimately developed pleural metastases. The primary tumor was assigned to WHO grade II, and the recurrent tumor to grade III. While this paper by itself does not provide truly novel information, in my view such rare patients should be reported in the literature. This and similar reports will help to build a database which may at some point help to improve our understanding and management of rare conditions. The report may benefit from a more comprehensive and detailed (tabular?) review of the literature.

I have read this submission. I believe that I have an appropriate level of expertise to confirm that it is of an acceptable scientific standard, however I have significant reservations, as outlined above.

# Response

**Published:** 2014-06-27

**Authors:** Suhail Basunaid

**Affiliations:** AZM/Maastricht University, Netherlands

I would like to thank Dr. Simon for his time and valuable comment. I wish to leave my current version as it is.

# Peer review: Referee response for version 1

**DOI:** 10.5256/f1000research.2443.r2143

**Published:** 2013-10-29

**Authors:** Daniel Vorobiof

**Affiliations:** 1 Department of Medical Oncology, Sandton Oncology Centre, Johannesburg, South Africa

This case report documents an unusual spread of a malignant meningioma. It doesn't add any further information and doesn't contribute any new knowledge, neither diagnostic nor therapeutic to the current available medical literature.

I have read this submission. I believe that I have an appropriate level of expertise to state that I do not consider it to be of an acceptable scientific standard, for reasons outlined above.
